# Preferences for TB treatment and support delivery models among people living with TB in Eastern Cape, South Africa: a discrete choice experiment

**DOI:** 10.1002/jia2.26506

**Published:** 2025-07-07

**Authors:** Michael Strauss, Gavin George, Emma Lansdell, Kuhle Fiphaza, Andrew Medina‐Marino, Joseph Daniels

**Affiliations:** ^1^ Health Economics and HIV and AIDS Research Division (HEARD) University of KwaZulu‐Natal Durban South Africa; ^2^ Division of Social Medicine and Global Health Lund University Lund Sweden; ^3^ Division of Men's Health Desmond Tutu HIV Centre University of Cape Town Cape Town South Africa; ^4^ Perelman School of Medicine University of Pennsylvania Philadelphia Pennsylvania USA; ^5^ Edson College of Nursing and Health Innovation Arizona State University Phoenix Arizona USA

**Keywords:** discrete choice experiment, preferences, tuberculosis, adherence, public health, Africa

## Abstract

**Introduction:**

South Africa has one of the highest incidence rates of notified tuberculosis (TB) in the world. Achieving TB control requires strengthening treatment and support services. The implementation of differentiated delivery models can be used to improve service quality and enhance retention in care. This study aimed to identify treatment and support delivery preferences among people on TB treatment, specifically examining gender differences, to inform the development of differentiated care models for improving engagement and retention in TB treatment services.

**Methods:**

A binary, unlabelled, fractional factorial design discrete choice experiment (DCE) was used to investigate preferences for TB treatment adherence support and service delivery. Attributes included who provides the support, how and where support is delivered, medication collection location and frequency of clinic visits. The DCE was administered to individuals who were currently on or recently completed TB treatment, and to those at‐risk for being lost‐to‐care. Data from 284 individuals for the DCE were collected from March to August 2022. Mixed effects logistic regression models were used as primary analysis tools. Latent class analysis (LCA) was used to explore heterogeneity in preference structures.

**Results:**

Compared to standard clinic‐based treatment collection, participants preferred collecting their treatment from a mobile community‐based location (ß = 0.231; 95% CI: 0.08–0.39), clinic‐based fast‐tracked pick‐ups (ß = 0.539; 95% CI: 0.38–0.70) or home delivery (ß = 0.563; 95% CI: 0.37–0.75). Participants also significantly preferred support offered monthly compared to once‐off (ß = 0.167; 95% CI: 0.01–0.32). Furthermore, participants preferred face‐to‐face support over group (ß = –0.142; 95% CI: –0.27 to –0.02) or phone‐based (ß = –0.222; 95% CI: –0.36 to –0.09) support models. LCA revealed three classes with statistically similar preference structures; Class 1 (62%) preferred community‐based treatment delivery and support services; Class 2 (28%) preferred clinic‐based support and treatment delivery services; and Class 3 (10%), preferred self‐selected peer navigator or nurse delivered, and group models of support and prioritised the location of medication pickups, with a preference for any model other than standard clinic collection.

**Conclusions:**

Though preference structures did not differ by gender, respondents revealed strong preferences for differentiated service delivery models. Future TB treatment and support interventions must include both clinic‐ and community‐based models of care and support to ensure that those living with TB are provided the greatest access to TB treatment and support services.

## 1 INTRODUCTION

In 2022, an estimated 280,000 individuals were diagnosed with tuberculosis (TB) in South Africa, and ∼54,000 people died of the disease [1]; of those who initiated TB treatment, only 78% successfully completed their treatment [2]. Poor adherence to TB treatment increases the risk of delayed culture conversion resulting in continued transmission, treatment failure, relapse, death and increases the risk of drug resistance [3]. Studies have shown that men are at increased risk for poor treatment outcomes due to a host of risk factors, including poor treatment adherence [4, 5]. Poor adherence to TB treatment increases morbidity and mortality among all people living with TB, which can lead to catastrophic economic costs [6]. To achieve the End TB Strategy target of halving TB incidence by 2025, and a 95% reduction in TB deaths by 2035, improving treatment adherence and retention in care is critical [7].

Interventions and services that cater for individual healthcare user preferences are more likely to be utilised and effective than those services simply designed based on the capacity of the health system and preferences of providers [8, 9, 10]. In this study, we used a discrete choice experiment (DCE) to analyse the preference structures of people on TB treatment [11, 12], to guide the development of differentiated care models for TB treatment and support. DCEs are increasingly used by health researchers to understand intervention preferences for the adaptation of existing interventions and service delivery models or for the development of new interventions [13, 14, 15]. Few DCEs have been conducted to assess TB treatment service and support preferences. One DCE conducted in Zambia revealed a preference for accessing TB diagnostic services close to home with short wait times [16]. A study in Uganda found a preference for treatment delivered by community health workers (CHWs) rather than a family member, home rather than workplace deliveries and monthly travel vouchers as a means of support rather than SMS reminders or phone calls [17]. We found no studies which sought to ascertain preferences for TB treatment service delivery models that evaluated differences across genders. However, research has shown that social and gender norms influence men's and women's health‐seeking behaviours, and their experience and perceptions of health facilities and healthcare providers—potentially requiring tailored health service models, if health outcomes are to be optimised [18, 19, 20, 21, 22, 23, 24, 25, 26, 27].

This study examines the preference structures of people on TB treatment for TB treatment and support service delivery models, including an analysis of gendered differences. The study further unpacks preference distinctions between people currently on TB treatment; those defaulting on treatment, and those who had successfully completed treatment. Evidence is expected to inform the development of differentiated care models with the aim of improving engagement and retention in TB treatment services.

## 2 METHODS

The DCE design focused on preferences for TB treatment and support delivery (primarily for adherence support, although we recognise that the nature of support people on TB treatment need may go beyond simply adherence). In addition, we analysed divergence in preferences to understand: (1) whether men and women have different priorities in terms of treatment and support delivery models; (2) whether people who successfully completed TB treatment have different preference structures from those who are at risk of being lost‐to‐care or those currently on treatment; and (3) whether there were unobserved sub‐populations within the sample that share similar preference structures supporting Information.

### 2.1 Study setting

Participants were recruited from 12 Primary Health Clinics in Buffalo City Metropolitan Health District, Eastern Cape Province, South Africa—a historically under‐resourced and high TB burden province (see Table 1).

**Table 1:** Buffalo City Metropolitan Health District [2, 28, 29, 30]

Population	794,324
Tuberculosis (TB) incidence	769/100,000
TB/HIV co‐infection rate	45.7%
Men:Women TB prevalence ratio	1.9:1
Drug‐sensitive TB treatment success rate	74.1%
TB patient loss‐to‐follow up	17.2%
TB patient death rate	7.9%

### 2.2 Study design

An initial list of 18 potential attributes (service delivery model characteristics) was developed based on findings from previous research [31, 32] and analysis of qualitative interviews conducted in a prior stage of the larger study [33]—additional results from the larger study are published elsewhere [33, 34, 35]. Following engagement with stakeholders, six attributes were included in the design to reduce the possibility of attribute non‐attendance [36] in two dimensions—adherence support delivery characteristics (frequency of support, who provides the support, how support is delivered, where support is delivered) and treatment delivery characteristics (medication collection location and frequency of clinic visits). The levels (possible options available for each attribute) for each attribute were determined based on currently available options and on analysis of qualitative data (Table 2).

**Table 2:** Final attributes and levels included in the discrete choice experiment design

Attribute	Levels			
Frequency of support	Once‐off^a^	Weekly	Monthly	As needed
Who provides the support	Peer navigator (self‐selected)^a^	Peer navigator (assigned)	Nurse	Community health worker (CHW)
How support is delivered	Individual face‐to face^a^	Small group	Individual phone	
Where support is delivered	Clinic‐based^a^	Home‐based	Community site	
Medication collection	Collection at the clinic (standard, longer waiting time)^a^	Collection at the clinic (fast track, shorter waiting time)	Delivery to your house	Pill collection at mobile/community site
Number of clinic visits	Weekly^a^	Once every 2 weeks	Once a month	Once every 2 months

^a^Levels used as the reference category for the analysis were selected as the attributes that were closest to “standard of care.”

The DCE used a binary choice, unlabelled, fractional factorial design generated in STATA17 to produce a statistically optimal design of 48 choice sets using the D‐efficiency criterion, based on a modified Federov algorithm [37, 38, 39]. Participants were each allocated a sub‐set of just 12 choice sets each. A blocking variable was used to divide the 48 choice sets into four blocks each with 12 choice sets—participants were randomly allocated to answer the choice sets in just one block [11].

### 2.3 Data collection

Data for the DCE were collected from March to August 2022. The fieldworker administered DCE was embedded in a larger quantitative survey which included basic socio‐demographic, behavioural measures and health history questionnaire. DCE choice sets were presented on electronic tablets using REDCap hosted at the University of KwaZulu‐Natal [40]. Before beginning the DCE, fieldworkers read scripted introductions, which included definitions of each attribute and level to ensure consistency in participants’ understanding (see File S1). For example, fast‐track clinic‐based models were explained as being based at a clinic but having separate queues, medication pick‐up points or patient flow procedures to speed up service delivery.

### 2.4 Sampling and eligibility criteria

Given the limited availability of prior estimates for sample size calculations for the DCE, we used a common convention to estimate a minimum sample size (*n*), where $n\geq 500\frac{L}{Sl}$, *L* is the maximum number of levels for any attribute (three), *S* is the number of choices in each choice set (two) and *J* is the number of tasks (or choices) presented to each participant (12) [41]. The minimum sample size for the DCE was 63 participants per stratification.

Participants were recruited based on: (1) gender; and (2) treatment group. Participants self‐identified as male or female in the survey; however, we have interpreted this data from a gender perspective, given that preferences are more likely to be associated with socially constructed roles and behaviours of men and women, rather than because of differences in biological sex. Treatment groups were defined as follows: (1) those currently on treatment; (2) those at risk of being lost‐to‐care (defined as having missed ≥ 15 days of a treatment refill visit); and (3) those who successfully completed treatment within 1 month of recruitment. Eligibility criteria: age ≥ 18 years; currently or recently engaged in TB treatment in one of the selected study clinics; able to speak English or isiXhosa; provided informed consent. Exclusion criteria: extra‐pulmonary TB without lung involvement; currently in the intensive phase of treatment; and positive smear grading at treatment initiation without a post‐intensive phase smear conversion to smear‐negative status.

### 2.5 Statistical analysis

A mixed effects logistic regression model was used as the primary analysis tool (Model 1), using dummy coding and Halton draws with 1000 replications to estimate the relative utility of each attribute level to understand overall preference structures. Mixed effects logit models allow for an initial assessment of heterogeneity in preferences across attributes by estimating both the mean utility and standard deviation [10]. Secondary analyses aimed to examine preference heterogeneity. First, we used mixed effects logistic regression models stratified by gender (Model 2) and then by treatment group (Model 3) and directly compared the ß‐coefficient estimates and 95% confidence intervals to understand divergence in preferences. Second, we used latent class models to further explore preference heterogeneity in the sample [10]. Latent class models assume that there are classes or segments within a sample that have similar preference weights that are systematically different from preferences in other classes that are linked to unobserved or unobservable participant characteristics [10]. These models are useful for understanding heterogeneity and how preferences cluster within sub‐groups of participants. We explored the model fit of four‐, three‐ and two‐class models. Goodness of fit was evaluated using the Akaike Information Criterion (AIC) and Bayesian Information Criterion (BIC) statistics, the mean predicted probability of class membership, and an examination of the size of the classes produced in each model. Correlations between Class membership and socio‐demographic characteristics were tested using Pearson's Chi‐squared test.

### 2.6 Ethics and integrity

Ethics approval was obtained from the Human Research Ethics Committee of the Faculty of Health Sciences, the University of Cape Town (Ref no.: 673/2019; Medina‐Marino, PI), with an institutional reliance agreement by Arizona State University (Daniels, PI). Study approval was provided by the Eastern Cape Provincial Department of Health (Ref no.: EC202010_023). All participants provided written informed consent and were provided a small snack and R50 (∼$3 USD) for their time. Study staff were trained in DCE administration, human subject's protection and good clinical practice.

## 3 RESULTS

### 3.1 Sample characteristics

We enrolled 284 participants (Median age = 40 years [IQR: 31–47 years]; *n* = 115 individuals currently on treatment; *n* = 90 who had recently completed treatment; and *n* = 79 at‐risk of being lost‐to‐care). Of those, 193 (66.09%) were men, 130 (50%) were living with HIV and 72 (25%) had a previous history of TB disease (Table 3).

**Table 3:** Participant socio‐demographic and clinical characteristics

	Currently on treatment (*n* = 115)	Recently completed (*n* = 90)	Stopping behaviour (*n* = 79)	Total (*N* = 284)
**Gender**	76 (66.09%)	59 (65.56%)	58 (73.42%)	193 (67.96%)
Men				
Women	39 (33.91%)	31 (34.44%)	21 (26.58%)	91 (32.04%)
**Median age (years), IQR**	42 (33–50)	40 (31–48)	35 (27–43)	40 (31–47)
**HIV status**				
HIV positive	55 (52.88%)	43 (53.09%)	32 (43.24%)	130 (50.19%)
HIV negative	49 (47.12%)	38 (46.91%)	42 (55.76%)	129 (49.81%)
**TB history**				
Never had TB	87 (41.23%)	70 (77.78%)	54 (69.23%)	211 (74.56%)
TB diagnosis < 2 years ago	7 (6.09%)	8 (8.89%)	11 (14.1%)	26 (9.19%)
TB diagnosis > 2 years ago	21 (18.26%)	12 (13.33%)	13 (16.67%)	46 (16.25%)
**Living status**				
Live alone	19 (16.52%)	9 (10.00%)	13 (16.67%)	41 (14.49%)
Live with someone	96 (83.48%)	81 (90.00%)	65 (83.33%)	242 (85.51%)
**Relationship status**				
Single	72 (62.62%)	50 (43.48%)	58 (50.43%)	180 (63.38%)
Married or living together	34 (29.57%)	33 (28.70%)	18 (15.65%)	85 (29.93%)
Separated/divorced/widowed	9 (7.83%)	7 (6.09%)	3 (2.61%)	19 (6.69%)
**Distance to clinic**				
Less than 1 km	31 (26.96%)	30 (33.33%)	25 (32.05%)	86 (30.39%)
1−2 km	31 (26.96%)	36 (40%)	32 (41.03%)	99 (34.98%)
3−5 km	31 (26.96%)	15 (16.67%)	8 (10.26%)	54 (19.08%)
More than 5 km	22 (19.13%)	9 (10%)	13 (16.67%)	44 (15.55%)
**Education**				
None	3 (2.61%)	5 (5.56%)	3 (3.85%)	11 (3.89%)
Grade 7 (primary school)	20 (17.39%)	6 (6.67%)	6 (7.69%)	32 (11.31%)
Grade 8−11 (secondary school)	64 (55.65%)	59 (65.56%)	48 (61.54%)	171 (60.42%)
Grade 12 (completed secondary school)	25 (21.74%)	16 (17.78%)	20 (25.64%)	61 (21.55%)
Tertiary	3 (2.61%)	4 (4.44%)	1 (1.28%)	8 (2.83%)
**Employment status**				
Working for pay	23 (20.00%)	19 (21.11%)	20 (25.64%)	62 (23.4%)
Homemaker/working without pay	4 (3.48%)	2 (2.22%)	1 (1.28%)	7 (2.64%)
Full‐time scholar or student	2 (1.74%)	3 (3.33%)	0 (0.00%)	5 (1.89%)
Long‐term sick/disabled/retired	11 (9.57%)	7 (7.78%)	3 (3.85%)	21 (7.92%)
Unemployed	66 (57.39%)	55 (61.11%)	49 (62.82%)	170 (64.15%)
Other	9 (7.83%)	4 (4.44%)	5 (6.41%)	18 (6.79%)
**Household income**				
Under R 2000 (< $133USD)	87 (75.65%)	62 (68.89%)	51 (65.38%)	200 (70.67%)
R 2000−R 5000 (≥ $133–$333USD)	22 (19.13%)	21 (23.33%)	22 (28.21%)	65 (22.97%)
R 5000−R 10,000 (≥ $333–$667USD)	4 (3.48%)	5 (5.56%)	5 (6.41%)	14 (4.95%)
More than R 10,000 (> $667USD)	2 (1.74%)	2 (2.22%)	0 (0%)	4 (1.41%)

*Note*: Categories that do not sum to 100% are due to missing data. Abbreviations: IQR, interquartile range; TB, tuberculosis.

### 3.2 DCE main effects

**Figure 1:**
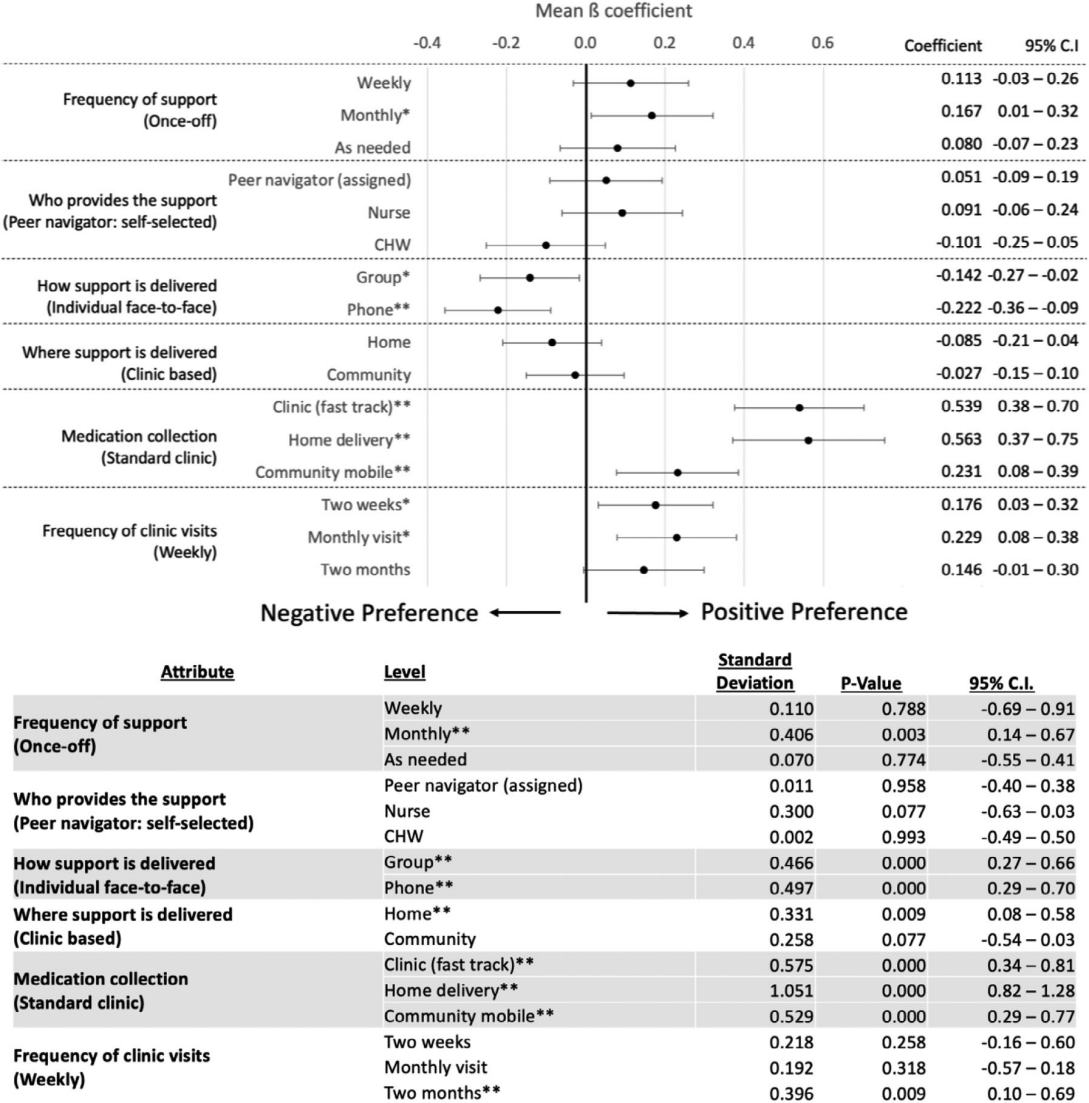
**Mixed effects logistic regression main effects**. **Top Figure**: Mean ß‐coefficients quantify the relative utility estimates, represented as point estimates. Error bars represent 95% confidence intervals. Numeric Mean ß‐coefficient estimates and 95% confidence intervals are shown on the right of the figure. **Bottom Table**: Standard deviation estimates for each of the mean ß‐coefficients along with 95% confidence intervals and *p*‐values. *Notes significant findings (*p*‐value < 0.05); **Notes highly significant findings (*p*‐value < 0.01); Abbreviations: CHW, community health worker; CI, confidence intervals.

Compared to once‐off support, participants had a significant preference monthly compared to once‐off support (ß = 0.167; 95% CI: 0.01–0.32), but no significant preference between support offered weekly or as needed. Similarly, participants revealed a relatively strong and significant preference for individual face‐to‐face support compared to group (ß = –0.142; 95% CI: –0.27 to –0.02) or phone‐based (ß = –0.222; 95% CI: –0.36 to –0.09) models of support. Participants did not reveal any significant preferences for who provides the support, or where the support is delivered (Figure 1).

Preferences for treatment delivery attributes were stronger than those for support delivery. Compared to standard clinic collection, participants had a slight preference for treatment pick‐ups from a mobile community‐based location (ß = 0.231; 95% CI: 0.08–0.39), and strong preferences for a fast‐track clinic‐based model (ß = 0.539; 95% CI: 0.38–0.70) and home delivery model (ß = 0.563; 95% CI: 0.37–0.75) for treatment collection. Participants also had a strong preference for monthly compared to weekly clinic visits (ß = 0.229; 95% CI: 0.08–0.38) and for visits every 2 weeks compared to weekly visits (ß = 0.176; 95% CI: 0.03–0.32).

Standard deviation estimates reveal significant divergence in preferences regarding where treatment (clinic fast‐track *p*‐value < 0.001; home delivery *p*‐value < 0.001; community mobile *p*‐value < 0.001) and support (home *p*‐value < 0.001) are delivered, how adherence support is delivered (group support *p*‐value < 0.001; phone‐based support *p*‐value < 0.001) and how frequently treatment (2 months *p*‐value = 0.009) and support (monthly *p*‐value = 0.003) are delivered. This finding of preference heterogeneity within the sample is explored more in the stratified analysis by gender and treatment group, as well as in the latent class analysis (LCA). The standard deviation estimates also suggest relatively consistent preferences for who provides the support, for frequency of support and for frequency of clinic visits (i.e. insignificant *p*‐values across most of the levels in these attributes).

### 3.3 Secondary analyses

#### 3.3.1 Preference structures by gender

**Figure 2:**
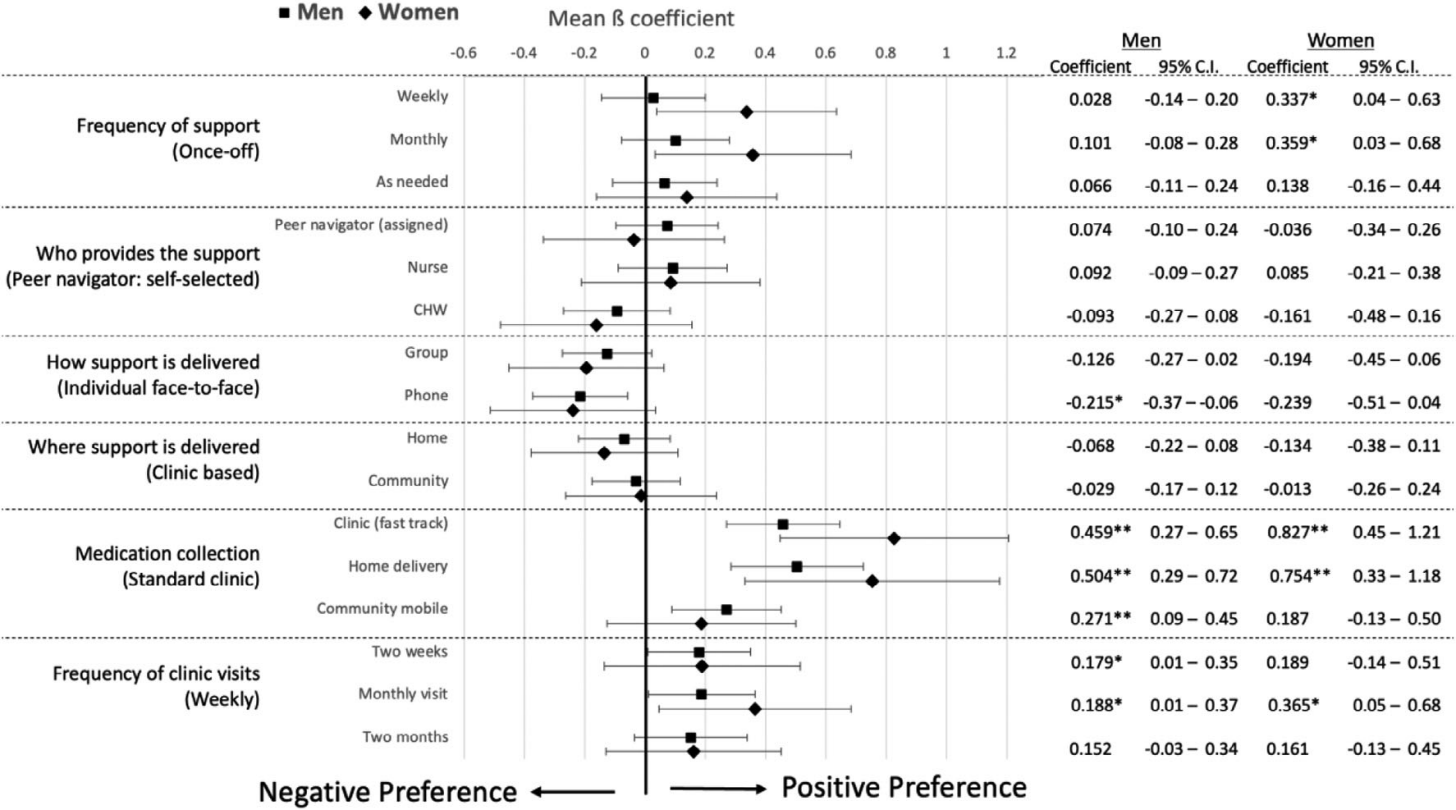
**Mixed effects logistic regression stratified by gender**. Mean ß‐coefficients quantify the relative utility estimates, represented as point estimates. Error bars represent 95% confidence intervals. Numeric Mean ß‐coefficient estimates and 95% confidence intervals are shown on the right of the figure. *Notes significant findings (*p*‐value < 0.05); **Notes highly significant findings (*p*‐value < 0.01). Abbreviation: CI, confidence intervals.

We found little divergence in preferences across gender (Figure 2). However, although the 95% confidence intervals are wide and overlapping, we note that while men seemed indifferent between alternatives for frequency of support, women had a significant preference for weekly (ß = 0.337; 95% CI: 0.04–0.63) or monthly support (ß = 0.359; 95% CI: 0.03–0.68) compared to once‐off support. Women also had a stronger preference than men for in‐clinic fast‐tracking (ß = 0.827; 95% CI: 0.45–1.21) and home delivery models of treatment collection (ß = 0.754; 95% CI: 0.33–1.18) compared to standard clinic‐based treatment collection. In addition, men's preference for face‐to‐face support compared to phone‐based models of support was significant (ß = –0.215; 95% CI: –0.37 to –0.06).

#### 3.3.2 Preference structures by treatment group

**Figure 3:**
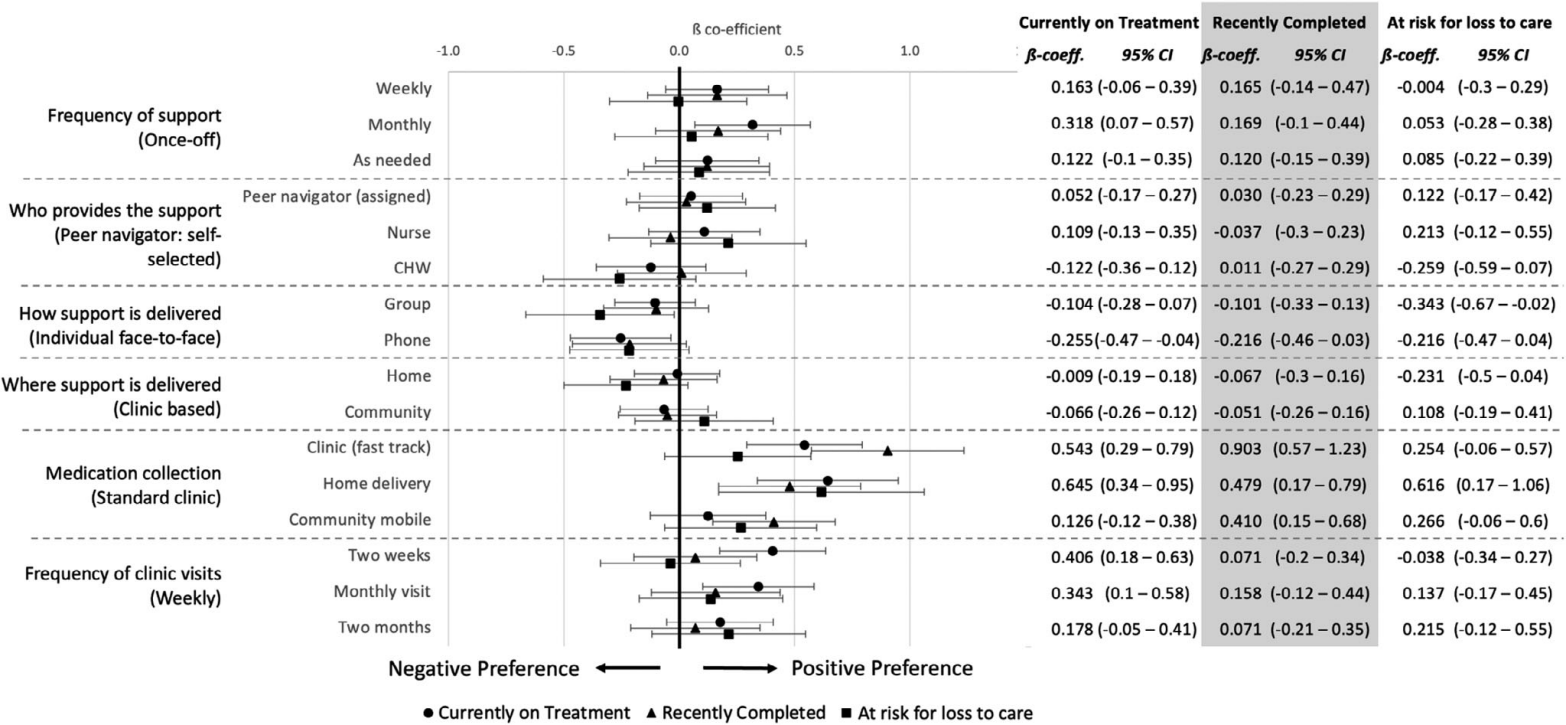
**Mixed effects logistic regression stratified by treatment group**. Mean ß‐coefficients quantify the relative utility estimates, represented as point estimates. Error bars represent 95% confidence intervals. Numeric Mean ß‐coefficient estimates, and 95% confidence intervals are shown on the right of the figure. Abbreviations: CHW, community health worker; CI, confidence intervals.

Preferences did not diverge substantially between treatment groups (Figure 3). However, some findings are worth noting.

##### 3.3.2.1 Frequency of support

Participants currently on treatment had a significant preference for monthly support (β = 0.318; 95% CI 0.07–0.57), while participants in the other two groups’ preferences regarding frequency of support were not significant.

##### 3.3.2.2 Who provides the support

There was no provider that participants significantly preferred to a self‐assigned peer navigator. Among participants at‐risk for loss‐to‐care, there is a statistically meaningful preference for a nurse compared to a CHW (95% CIs do not overlap ß‐coefficient estimates).

##### 3.3.2.3 How support is delivered

First, participants at‐risk for being loss‐to‐care had a significant preference for individual face‐to‐face support compared to group‐based models of support (β = –0.343; 95% CI: –0.67 to –0.02); this was not significant for participants in the other two groups. Second, participants currently on treatment expressed a significant preference for individual face‐to‐face support compared to phone‐based support (β = –0.225; 95% CI: –0.47 to –0.04); this was not significant in the other two groups.

##### 3.3.2.4 Medication collection

First, participants currently on treatment (β = –0.543; 95% CI: 0.29–0.79) and those who recently completed treatment (β = –0.903; 95% CI:0.57–1.23) had a stronger preference for clinic‐based fast‐track medication collection compared to standard clinic collection; this was not significant for participants at‐risk for being loss‐to‐care. Second, participants who had recently completed treatment had a significant preference for community mobile medication collection compared to standard clinic collection (β = –0.41; 95% CI: 0.15–0.68); this was not significant for participants in other groups. Third, all participant groups had a significant preference for home delivery of treatment compared to standard clinic collection of their medication.

##### 3.3.2.5 Frequency of clinic visits

Participants currently on treatment showed a significant preference for less frequent clinic visits (β = –0.225; 95% CI: –0.47 to –0.04); this was not significant for participants in other groups.

#### 3.3.3 Latent class analysis

Three latent class models were explored (with two, three and four classes, respectively). The mean predicted probability of all three models was high, and the AIC and BIC measures for model fit did not suggest an objectively better fit of any of the three models. The three‐class model fit the data best based on the log‐likelihood and BIC criterion and had a high mean probability of class membership (see Table 4).

**Table 4:** Latent class model fit statistics

	Number of observations	Log‐likelihood	Akaike Information Criterion	Bayesian Information Criterion	Mean probability of class membership
Two‐class model	6816	−2242.8521	4551.7038	4672.12	0.9744865
Three‐class model	6816	−2213.3963	4526.7921	4709.2408	0.8750903
Four‐class model	6816	−2193.74	4521.48	4765.9613	0.8727274

The two‐ and four‐class models produced very small class sizes for all but one of the classes. We thus present results from the three‐class model (Table 5). Results from the two‐ and four‐class models are presented in Supporting information File S2. We found no significant correlations between socio‐demographic characteristics and class membership.

**Table 5:** Latent class analysis results for a three‐class model

Attribute (reference category)	Level	Class 1 (*n* = 176) Coeff. (SE)	Class 2 (*n* = 80) Coeff. (SE)	Class 3 (*n* = 28) Coeff. (SE)
**Frequency of support** (Once‐off)	Weekly	0.106 (0.09)	0.127 (0.18)	−2.503 (1.07)^b^
	Monthly	0.267 (0.11)^b^	−0.01 (0.20)	−4.292 (2.01)^b^
	As needed	0.210 (0.09)^b^	−0.17 (0.17)	−2.375 (1.30)^a^
**Who provides the support** (Peer navigator: self‐selected)	Peer navigator	0.023 (0.09)	0.088 (0.17)	−2.405 (1.15)^b^
	Nurse	−0.027 (0.09)	0.250 (0.17)	1.507 (1.00)
	CHW	−0.173 (0.10)^a^	0.073 (0.19)	−1.034 (1.02)
**How support is delivered** (Individual face‐to‐face)	Group	−0.205 (0.08)^b^	0.024 (0.14)	2.442 (1.05)^b^
	Phone	−0.07 (0.08)	−0.375 (0.15)^c^	−2.93 (1.41)^b^
**Where support is delivered** (Clinic‐based)	Home	0.194 (0.09)^b^	−0.696 (0.21)^c^	−1.125 (1.05)
	Community	0.089 (0.08)	−0.327 (0.18)^a^	−1.049 (0.84)
**Medication collection** (Standard clinic)	Clinic (fast‐track)	0.348 (0.1)^a^	0.296 (0.17)^a^	9.899 (3.84)^c^
	Home delivery	0.627 (0.13)^a^	−0.701 (0.21)^c^	14.092 (4.84)^c^
	Community mobile	0.327 (0.11)^a^	−0.425 (0.18)^b^	7.665 (2.74)^c^
**Frequency of clinic visits** (Weekly)	Two weeks	0.071 (0.09)	0.226 (0.16)	4.171 (1.77)^b^
	Monthly visit	0.265 (0.09)^a^	−0.056 (0.19)	−0.080 (0.83)
	Two months	0.117 (0.10)	0.043 (0.20)	0.672 (0.56)

Abbreviations: CHW, community health worker; SE, standard error.

^a–c^ Significance at 10% (a), 5% (b) and 1% (c) levels.

Class 1 contained 176 (61.97%) participants, characterised by a preference for home‐based compared to clinic‐based support, and for home or community mobile delivery of treatment compared to standard clinic medication collection. Participants in this class also preferred clinic‐based fast‐tracking of medication collection compared to standard clinic‐based medication collection. Participants in Class 1 had a significant preference for monthly compared to weekly clinic visits, and monthly support or support provided as needed compared to once‐off support. Participants in Class 1 preferred not to have support delivered by CHWs compared to a self‐selected peer navigator, and preferred not to have support delivered using group models, compared to individual face‐to‐face support.

Class 2 contained 80 (28.17%) participants, characterised by a preference for clinic‐based support compared to support delivered at home or in the community, and for medication delivered to their home or picked up in the community compared to standard clinic‐based medication collection. These participants had a significant preference for clinic‐based fast‐tracking of medication collection compared to standard clinic‐based medication collection. Participants in Class 2 had a significant preference for individual face‐to‐face support compared to support delivered by phone.

Class 3 contained 28 (9.86%) participants, characterised by a preference for home delivery of medication, community mobile medication collection and a fast‐track clinic‐based model for medication collection compared to the standard clinic‐based model. There was also a significant preference for once‐off support compared to monthly, weekly or as‐needed support. Class 3 participants also preferred: (1) a self‐selected versus an assigned peer navigator to deliver support to them; (2) group models of support compared to individual face‐to face support; and (3) not to have support delivered by phone compared to individual face‐to‐face support. Finally, there was a preference in this class for clinic visits every 2 weeks compared to clinic visits every week.

## 4 DISCUSSION

This is one of the first studies using a DCE to identify the preferences of people on TB treatment for service delivery models of TB treatment and support interventions in South Africa. Study participants prioritised treatment service delivery model characteristics above adherence support model characteristics. Participants preferred less frequent clinic visits, and fast‐track medication pick‐up, community‐based or home delivery of treatment were favoured over standard clinic medication pick‐up, a finding consistent with global guidance for designing differentiated models of care [42]. Fast‐track clinic‐based models for providing differentiated treatment services have been applied in different ways for HIV treatment, but share the same broad characteristic of creating separate queues, medication pick‐up points or patient flow procedures at a health facility to speed up service delivery for stable patients [43]. However, these models have not yet been systematically developed or tested for stable TB patients. There was no clear preference for who (i.e. peer navigator, nurse or CHW) provided treatment support, and participants expressed preference for individual, face‐to‐face support rather than group‐ or phone‐based support.

Our findings suggest that preferences for TB treatment service delivery in South Africa may not vary substantially across gender. However, preferences are likely to have distinct gendered motivations, and previous research suggests that beyond service delivery model characteristics, the content and focus of the support may need to be responsive to men's and women's specific needs. For example, masculinity conventions and social structures can affect how men interpret the severity of their illness, influencing their healthcare behaviour [19]. Further, pervasive social expectations, including viewing men's primary role as family provider, can restrict their optimal treatment adherence and retention in care [34, 44]. For women, the perceived stigma associated with TB illness and treatment will need to be addressed to ensure care is sought [45, 46, 47, 48].

The analysis of preference structures by treatment group suggests that preference structures were mostly consistent across groups, with a few notable exceptions. Participants at‐risk for being lost‐to‐care had a stronger preference for individual face‐to‐face support over group‐ or phone‐based models, and for support delivered by a peer navigator (either self‐selected or assigned) rather than a CHW. In addition to these service delivery model considerations, efforts to reduce stigma and strengthen social support networks, mental resources and health services for people on TB treatment are likely to be equally important in subsequent intervention design [4, 34, 35].

The LCA revealed two dominant groupings of participants with similar preference structures—those who prefer community‐based services (Class 1) and those who prefer clinic‐based services (Class 2). A small percentage (Class 3—10%) preferred any treatment delivery option, other than standard clinic collection and community‐based support. Furthermore, home‐based support provided by peer navigators or nurses was preferred to support provided by CHWs. Such preferences align with studies reporting that community‐based models of care could help reduce the burden on health facilities and improve the convenience of medication pick‐up [49]. Our findings differed from those found in a DCE conducted in Uganda where people living with multi‐drug resistant TB support to be provided by a CHW [17].

Our findings show that community‐based services are not preferred by all participants and that some participants prefer clinic‐based services, both in terms of medication collection and support. In particular, fast‐track medication collection was preferred, highlighting the importance of improving and streamlining processes for TB treatment and support at the clinic level.

The treatment of TB does not occur within a vacuum, with substantial evidence on the linkages between HIV and TB, and a higher likelihood of mortality among people living with co‐infections [50]. A consolidation of findings across studies examining preferences for differentiated models of care for both HIV and TB may be useful for the design of TB interventions and integrated services. HIV treatment studies have found a preference for less frequent visits and shorter wait times [51, 52, 53], individual adherence support [52] and fast‐track health facility‐based models over home‐delivery or community‐based models [51, 53]. Several HIV studies have also shown little difference in preference structures across gender [51, 52, 54].

Our study has several limitations. First, the limited geographic scope of the study limits its generalisability to other contexts. The small sample size in each treatment group may have prevented us from identifying all statistical differences in preferences. However, based on our results, the magnitude of these differences is likely to remain small, even if statistical significance could be improved. Further, differences in preferences may not be driven solely or primarily by gender or treatment outcomes. Although we conducted a substantial exploration of the correlation between the latent classes that emerged and socio‐demographic characteristics of interest (including treatment group and gender), we found no significant correlations. This may be related to the small sample, or to complex interactions between participant characteristics (both observed and unobserved) that drive preferences. We leave for future research the task of linking participant characteristics to latent class groupings.

Third, participants were not eligible for inclusion if they were still engaged in the intensive phase of treatment meaning we could not capture preferences of people on TB treatment in this early phase, or those lost from care before the continuation phase of treatment. The data are also cross‐sectional so we are unable to capture how preferences change over time.

Finally, our DCE focused on the characteristics of service delivery models for TB treatment support rather than the characteristics of the types or content of the support—a design decision based on the research question. Further investigation is needed to understand how participants value and prioritise different types of support content.

## 5 CONCLUSIONS

One‐size‐fits‐all interventions for TB treatment service delivery and adherence support will not work for all people on TB treatment in South Africa. Both clinic‐based and community‐based models of care and support require strengthening. Improving differentiated models for medication collection and tailoring the frequency of clinic visits to individual preferences is key for improving treatment outcomes. In recognition of the high prevalence of TB‐HIV co‐morbidity, the evidence generated from this study provides valuable insights into the design and delivery of differentiated and integrated TB‐HIV services [55]. While this will require significant investment, the long‐term returns will be high in terms of better health, quality of life and economic productivity in a setting where HIV and TB prevalence remain high.

## COMPETING INTERESTS

The authors declare no conflicts of interest.

## AUTHORS’ CONTRIBUTIONS

MS, GG, AM‐M and JD conceptualised and designed the study. MS, GG, AM‐M, JD and KF developed the DCE instrument and KF and EL oversaw data collection, extraction and validation. MS and EL conducted the formal analysis and all authors provided critical input for the interpretation of findings and contextualisation of results. MS, GG and JD produced the first draft, and all authors contributed critical input for finalizing the manuscript. All authors have read and approved the final manuscript.

## FUNDING

The study was funded by the U.S. National Institutes of Health (NIH) under award R21AI148852 to AM‐M and JD, with additional support provided by NIH awards R34HL170819 to AM‐M and JD and R01AI150485 to AM‐M.

## ETHICS APPROVAL STATEMENT

Ethics approval was obtained from the Human Research Ethics Committee of the Faculty of Health Sciences, the University of Cape Town (Ref no.: 673/2019; Medina‐Marino, PI), with an institutional reliance agreement by Arizona State University (Daniels, PI). Study approval was provided by the Eastern Cape Provincial Department of Health (Ref no.: EC202010_023).

## PATIENT CONSENT STATEMENT

All participants provided written informed consent. Participants were provided a small snack and R50 (∼$3 USD) for their time. Study staff were trained in DCE administration, human subject's protection and good clinical practice.

## MATERIAL FROM OTHER SOURCES

No material from other sources was used in this manuscript.

## Supporting information




**Supporting file 1**: Introduction to DCE Questions.

**Supporting file 2**: 2‐class and 4‐class solutions for the latent class analysis.

## Data Availability

The data used for the analysis presented in this manuscript will be made available by the study PI on request.
