# Supplementary material for: Preferences for TB treatment and support delivery models among people living with TB in Eastern Cape, South Africa: a discrete choice experiment
Source: J Int AIDS Soc. 2025 Jul 7;28(Suppl 3):e26506. doi: 10.1002/jia2.26506 (PMC12232482; doi:10.1002/jia2.26506)
Supplement: Supplementary file 1 — Supporting file 1: Introduction to DCE Questions. [file JIA2-28-e26506-s001.pdf]

<u>Introduction to DCE Questions</u>

In the next section of the questionnaire, we are going to ask you to make some choices about the type of TB treatment and support services you would most prefer. In each choice, we will show you two different possible options for you to choose between. Each option is based on different possible combinations of characteristics of TB treatment services that you could get. Based on your experience of TB treatment and support, we want to know which types of services you think are best and would most prefer.

When you make the choices, you will see that some characteristics about ***treatment support***, and some of the characteristics are about the ***actual TB treatment***. TB support can be very important for helping people to complete their treatment properly and can focus on what people on TB treatment are most likely to need, such as: treatment adherence support, mental health support, support to deal with social stigma or anything else you might need in relation to the treatment program (we want you to think about the support you might need other than money or food). The treatment support characteristics we want you to think about are:

1. Who provides the support
2. Where the support is delivered
3. How the support is delivered
4. The frequency of support (how often you get support)

Who provides the support

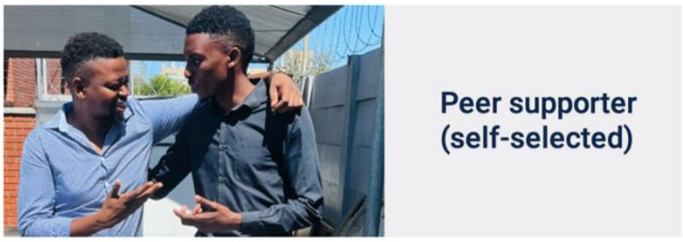

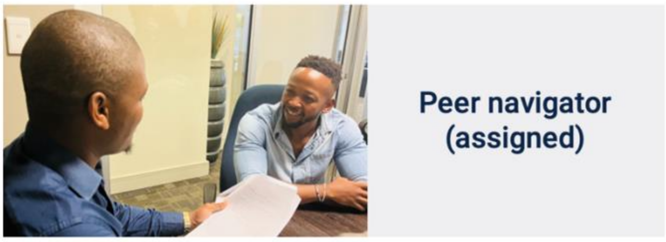

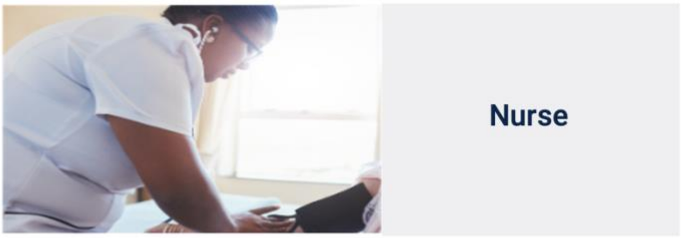

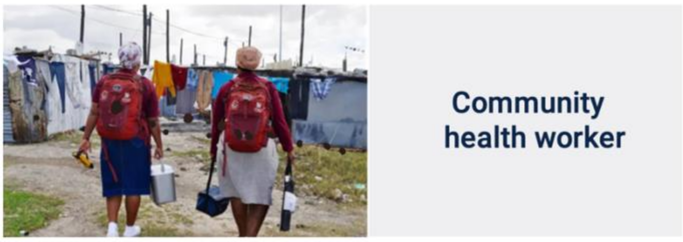

A peer navigator is usually a person who has had TB before, and has successfully completed their treatment, and who shares similar experiences to you and lives in the same community as the people they support. Peer navigators are trained to counsel, educate and motivate treatment and can provide support.

Self-selected peer supporter: A self-selected peer supporter is a person you would choose to be your supporter, from someone in your own network of friends and family. They would undergo a short training for supporting you through your treatment.

Assigned peer navigator: This would be a peer navigator assigned to you at the healthcare facility. It would be somebody who the clinic has already trained and has said they would be willing to help other people who are on TB treatment.

Where the support is delivered

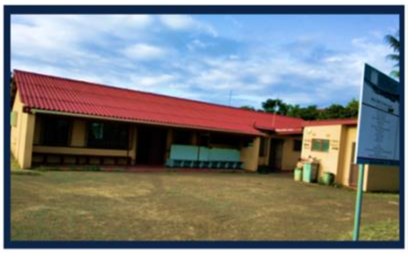

Clinic based

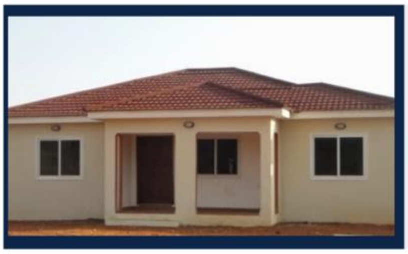

Home based

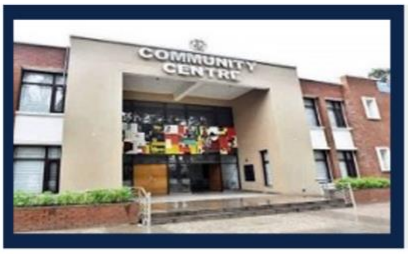

Community site

This could include venues like a nearby school, community hall, church or other suitable venue in your community

How the support is delivered

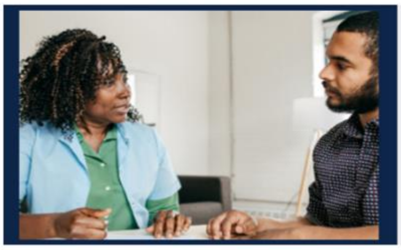

Individual face-to-face

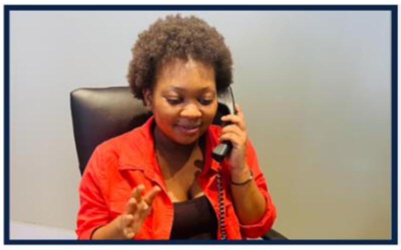

Individual phone

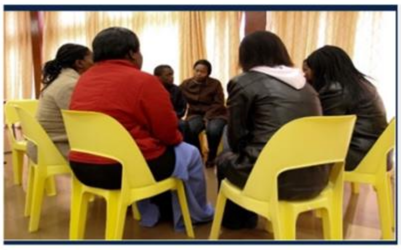

Small group

Between three and five other people in the group who also have TB and are currently on treatment

Frequency of support

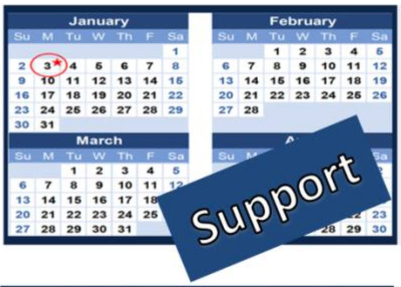

Once-off

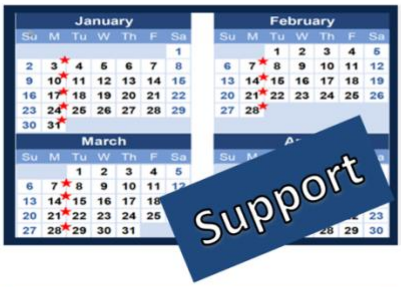

Weekly

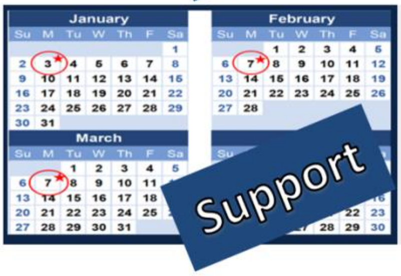

Monthly

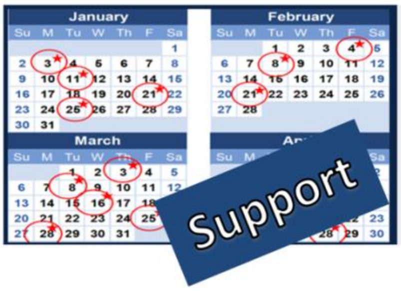

As needed

The TB treatment characteristics we want you to think about are focused on service delivery, and include:

1. Where you collect your medication from
2. How often you get pill refills

Medication collection

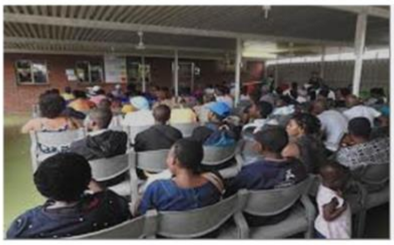

Standard clinic collection (longer waiting time)

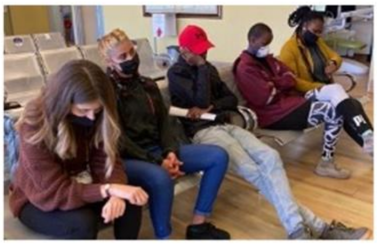

Fast track clinic collection (shorter waiting time)

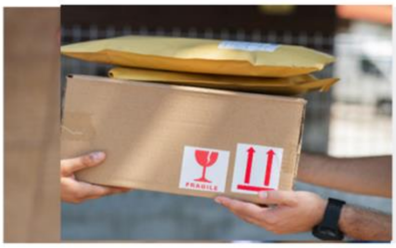

Delivery to your house

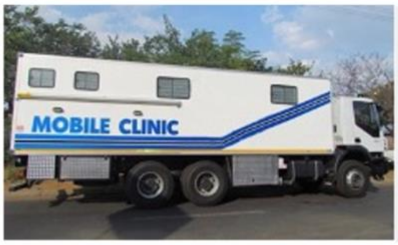

Pill collection at mobile/ community site

How often you get pill refills

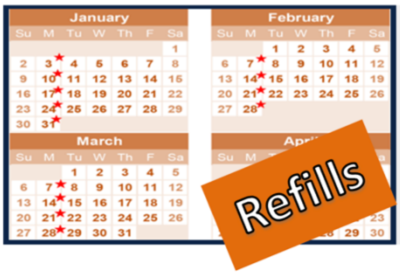

Once every week

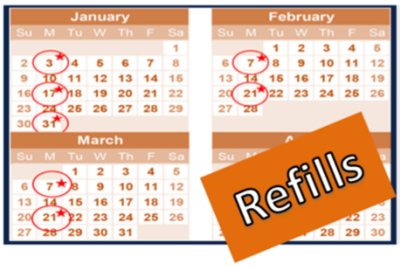

Once every two weeks

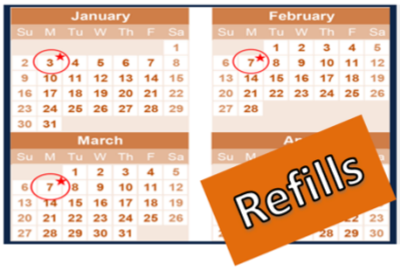

Once a month

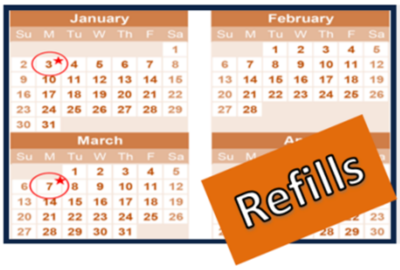

Once every two months

When you make your choices about which options you most prefer, you should try to think about which characteristics are most important to you, and this should influence the choice you make, especially if you think the two options are quite similar. If you like both options, choose one you prefer more, and if you don’t like either option, try to choose the one that you dislike the least. You should only choose one option for each question. You can assume that all other features that are not presented are otherwise the identical across the two programs.

<u>**Let’s have a look at an example:**</u>

	Option A		Option B	
**Frequency of support**	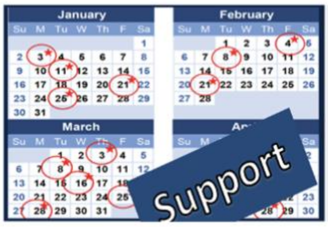	As needed	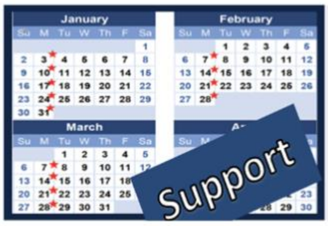	Weekly
**Who provides the support**	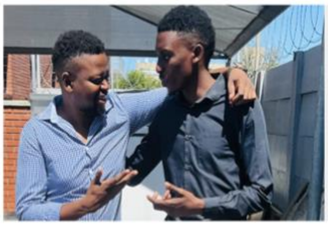	Peer supporter (self-selected)	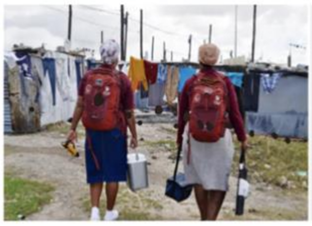	Community health worker
**How the support is delivered**	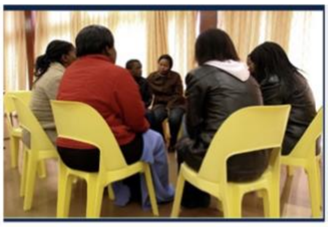	Small group	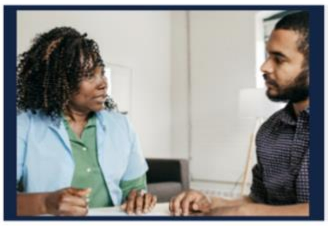	Individual face-to-face
**Where the support is delivered**	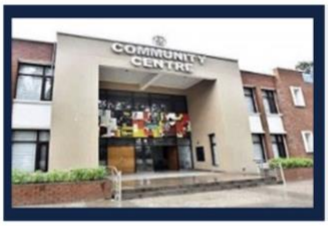	Community site	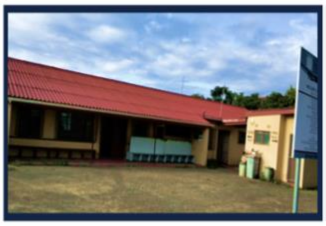	Clinic based
**Where you collect your TB medication**	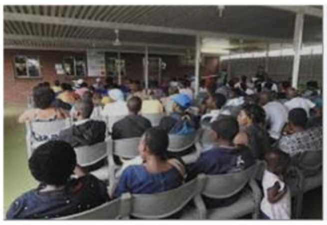	Standard clinic collection (longer waiting time)	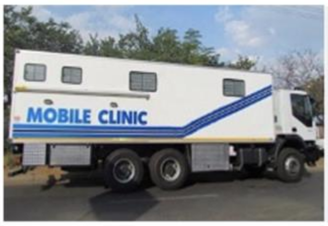	Pill collection at mobile/ community site
**How often you get your pill refills**	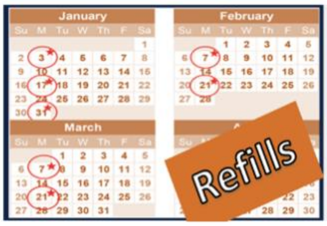	Once every two weeks	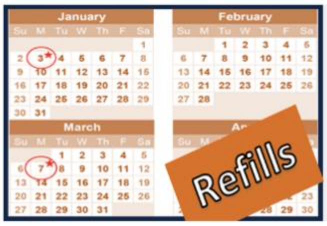	Once every two months

<u>Do you have any questions?</u>
