# Supplementary material for: Preferences for TB treatment and support delivery models among people living with TB in Eastern Cape, South Africa: a discrete choice experiment
Source: J Int AIDS Soc. 2025 Jul 7;28(Suppl 3):e26506. doi: 10.1002/jia2.26506 (PMC12232482; doi:10.1002/jia2.26506)
Supplement: Supplementary file 2 — Supporting file 2: 2‐class and 4‐class solutions for the latent class analysis. [file JIA2-28-e26506-s002.pdf]

2-class and 4-class solutions for the latent class analysis

2-class solution

```
lclogit choice $exvar, group(identifier) id(uniqueid) ncl(2)

Iteration 0:  log likelihood = -2304.3035
Iteration 1:  log likelihood = -2302.3809
Iteration 2:  log likelihood = -2297.3773
Iteration 3:  log likelihood = -2285.6605
Iteration 4:  log likelihood = -2269.6373
…
Iteration 92:  log likelihood = -2242.8533
Iteration 93:  log likelihood = -2242.8527
Iteration 94:  log likelihood = -2242.8524
Iteration 95:  log likelihood = -2242.8521

Latent class model with 2 latent classes

-------------------------------
    Variable |  Class1   Class2
-------------+-----------------
      weekly |  -0.407    0.106
     monthly |  -0.634    0.179
   as_needed |   0.304    0.095
peer_assig~d |  -0.340    0.039
       nurse |   0.909    0.074
         chw |  -0.218   -0.089
       group |   0.401   -0.116
       phone |  -0.303   -0.165
        home |   0.252   -0.097
   community |  -0.036   -0.049
clinic_fas~k |   2.908    0.294
home_deliv~y |   5.875    0.100
community_~e |   2.625    0.040
   two_weeks |   0.319    0.120
monthly_vi~t |   0.386    0.145
  two_months |   0.846    0.067
-------------+-----------------
 Class Share |   0.128    0.872
-------------------------------

Note: Model estimated via EM algorithm

. scalar N_2=e(N)
. scalar N_g_2=e(N_g)
. scalar ll_2=e(ll)
. scalar aic_2=e(aic)
. scalar bic_2=e(bic)
. scalar caic_2=e(caic)
. display N_2 ", " N_g_2 ", " ll_2 ", " aic_2 ", " bic_2 ", " caic_2
6816, 3408, -2242.8519, 4551.7038, 4672.12, 4705.12
```

4-class solution

```
lclogit choice $exvar, group(identifier) id(uniqueid) ncl(4)

Iteration 0:  log likelihood = -2296.296
Iteration 1:  log likelihood = -2282.5715
```

```
Iteration 2:  log likelihood = -2261.1624
Iteration 3:  log likelihood = -2242.1774
Iteration 4:  log likelihood = -2231.7661
Iteration 145:  log likelihood = -2193.8309
Iteration 146:  log likelihood = -2193.8127
Iteration 147:  log likelihood = -2193.795
Iteration 148:  log likelihood = -2193.7774
Iteration 149:  log likelihood = -2193.7592
Iteration 150:  log likelihood = -2193.74

Latent class model with 4 latent classes

-------------------------------------------------
    Variable |  Class1   Class2   Class3   Class4
-------------+-----------------------------------
      weekly |   0.093   -0.530    1.862    0.043
     monthly |   0.188   -0.842    2.047   -0.348
   as_needed |   0.109    0.298    0.332    0.007
peer_assig~d |   0.040   -0.322   -1.075    0.178
       nurse |   0.032    0.880    0.779    0.020
         chw |  -0.193   -0.201    1.327   -0.099
       group |  -0.173    0.570   -0.112    0.046
       phone |  -0.069   -0.425   -2.079   -0.234
        home |   0.054    0.159   -2.288   -0.095
   community |  -0.011    0.042   -1.420    0.302
clinic_fas~k |   0.166    3.245    0.664    1.067
home_deliv~y |   0.304    6.044   -0.665   -0.459
community_~e |   0.233    2.734   -0.140   -0.905
   two_weeks |   0.132    0.468    0.073    0.053
monthly_vi~t |   0.158    0.362   -0.766    0.453
  two_months |   0.027    0.955   -0.592    0.494
-------------+-----------------------------------
 Class Share |   0.642    0.124    0.082    0.152
-------------------------------------------------

Note: Model estimated via EM algorithm

. scalar N_4=e(N)
. scalar N_g_4=e(N_g)
. scalar ll_4=e(ll)
. scalar aic_4=e(aic)
. scalar bic_4=e(bic)
. scalar caic_4=e(caic)
. display N_4 ", " N_g_4 ", " ll_4 ", " aic_4 ", " bic_4 ", " caic_4
6816, 3408, -2193.74, 4521.48, 4765.9613, 4832.9613
```
